# A type 1 diabetes genetic risk score can discriminate monogenic autoimmunity with diabetes from early-onset clustering of polygenic autoimmunity with diabetes

**DOI:** 10.1007/s00125-018-4551-0

**Published:** 2018-02-07

**Authors:** Matthew B. Johnson, Kashyap A. Patel, Elisa De Franco, Jayne A. L. Houghton, Timothy J. McDonald, Sian Ellard, Sarah E. Flanagan, Andrew T. Hattersley

**Affiliations:** 1 Institute of Biomedical and Clinical Science, University of Exeter Medical School, Exeter, EX2 5AD UK; 2 Molecular Genetics, Royal Devon and Exeter Hospital, Exeter, UK

**Keywords:** Gene discovery, Genetic risk score, Monogenic autoimmune diabetes, Type 1 diabetes

## Abstract

**Aims/hypothesis:**

Identifying individuals suitable for monogenic autoimmunity testing and gene discovery studies is challenging: early-onset type 1 diabetes mellitus can cluster with additional autoimmune diseases due to shared polygenic risk and islet- and other organ-specific autoantibodies are present in both monogenic and polygenic aetiologies. We aimed to assess whether a type 1 diabetes genetic risk score (GRS) could identify monogenic autoimmune diabetes and be useful to prioritise individuals for gene discovery studies.

**Methods:**

We studied 79 individuals with diabetes and at least one additional autoimmune disease diagnosed before the age of 5 years. We screened all participants for the seven genes known to cause monogenic autoimmunity that can include diabetes (*AIRE*, *IL2RA*, *FOXP3*, *LRBA*, *STAT1*, *STAT3*, *STAT5B*). We genotyped the top ten risk alleles for type 1 diabetes, including HLA and non-HLA loci, to generate a type 1 diabetes GRS.

**Results:**

Of the 79 individuals studied, 37 (47%) had mutations in the monogenic autoimmunity genes. The type 1 diabetes GRS was lower in these individuals than in those without mutations in these genes (median 9th vs 49th centile of type 1 diabetes controls, *p* < 0.0001). Age of diabetes diagnosis and type 1 diabetes GRS combined to be highly discriminatory of monogenic autoimmunity (receiver operating characteristic AUC: 0.88). Most individuals without a mutation in a known gene had a high type 1 diabetes GRS, suggesting that they have polygenic clustering of type 1 diabetes and additional autoimmunity and should not be included in gene discovery studies.

**Conclusions/interpretation:**

We have shown that the type 1 diabetes GRS can identify individuals likely to have monogenic autoimmunity, helping both diagnostic testing and novel monogenic autoimmunity gene discovery. Individuals with monogenic autoimmunity have a different clinical course to those with polygenic type 1 diabetes and can respond well to therapies targeting the underlying genetic defect.

**Electronic supplementary material:**

The online version of this article (10.1007/s00125-018-4551-0) contains peer-reviewed but unedited supplementary material, which is available to authorised users.


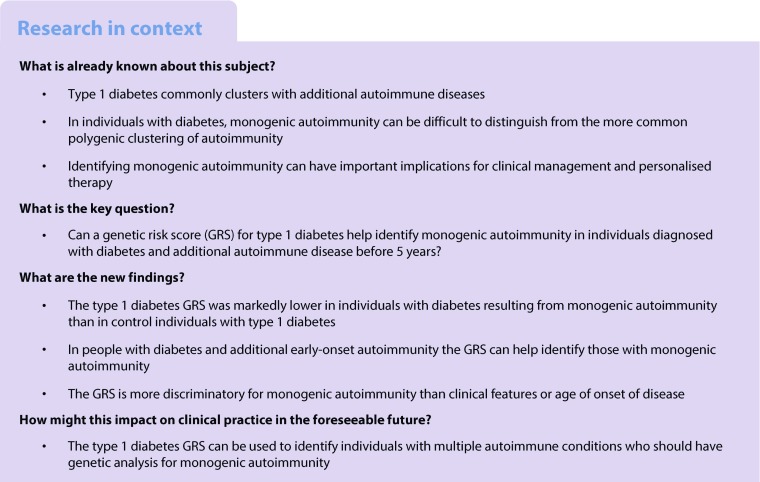


## Introduction

Monogenic autoimmune disease often presents with very-early-onset diabetes. For example, hemizygous mutations in *FOXP3* cause immunodysregulation, polyendocrinopathy, enteropathy, X-linked (IPEX) syndrome, which presents in the neonatal period with diabetes, protein-losing enteropathy and severe eczema [1]. Similarly, individuals with infantile-onset multisystem autoimmune disease due to dominant gain-of-function *STAT3* mutations or common variable immunodeficiency 8 with autoimmunity due to recessively inherited *LRBA* mutations may present with neonatal diabetes [2, 3].

While some individuals harbour a causative mutation in a single gene, the clustering of very-early-onset diabetes with autoimmune disease is usually due to a strong polygenic risk resulting from shared predisposing genetic loci. It is well established that the *HLA-DR3* haplotype is associated with the development of type 1 diabetes mellitus [4] and coeliac disease through its strong linkage with the *HLA-DQ2* haplotype [5]. Outside the HLA region the *IL2RA* polymorphism rs706778 is associated with increased risk of type 1 diabetes, autoimmune thyroid disease (AITD) and coeliac disease, as well as other paediatric-onset autoimmune disorders [6].

The phenotypic overlap between the two groups means that identifying individuals for testing is difficult using clinical features or biomarkers. While islet autoantibodies are highly discriminatory of type 1 diabetes against type 2 diabetes and MODY [7, 8], they are often present in individuals with monogenic autoimmunity. For example, multiple islet autoantibodies are present in more than half of individuals with IPEX syndrome [9]. Moreover, as it is thought that these individuals have autoimmune destruction of the pancreatic beta cells [10], serum C-peptide levels and treatment type or dose is also likely to be similar in the two groups.

The type 1 diabetes genetic risk score (GRS) is calculated by genotyping the top risk alleles and summing their effective weight to assign a numerical score to the individual that can be compared with control samples [11]. It was recently shown to be highly discriminatory of non-autoimmune monogenic diabetes and type 2 diabetes from type 1 diabetes [11, 12]. We sought to determine whether the type 1 diabetes GRS could distinguish between monogenic autoimmunity and polygenic clustering of autoimmune disease.

## Methods

### Study cohort

#### Individuals with early-onset autoimmunity

We studied 79 individuals diagnosed with autoimmune diabetes and one or more additional autoimmune disorder before the age of 5 years referred to the Exeter Molecular Genetics laboratory between 2005 and 2017 (Table 1). All individuals had previously been screened for all known monogenic diabetes genes [13]. Clinical information was supplied by the referring clinician from the person’s medical notes. All individuals had received a diagnosis of autoimmune diabetes from their clinician prior to genetic testing. Informed consent was obtained for all adult study participants and informed parental consent was given on behalf of children.

**Table 1:** Summary of the main clinical and demographic features of the cohort

Clinical/demographic feature	Monogenic autoimmunity (*n* = 37)	Unknown aetiology (*n* = 42)	*p* value
Consanguineous^a^	19/37 (51)	11/42 (26)	0.04
Sex, male:female ratio^b^	31:6	25:17	0.03
Diabetes characteristics			
Age at diagnosis, weeks	5 (0–83)	36 (1–258)	<0.001
Insulin dose, U kg^−1^ day^−1^	1.0 (0.6–1.2)	0.8 (0.5–1.1)	0.33
Islet autoantibody status (*n* = 43)			
Positive for ≥1 antibody	8/18 (44)	11/25 (44)	1.00
GAD positive	5/18 (28)	8/25 (32)	1.00
IA2 positive	2/18 (11)	2/25 (8)	1.00
ICA positive	2/18 (11)	3/25 (12)	1.00
ZnT8 positive	1/18 (5)	0/25 (0)	0.42
Additional autoimmune diseases			
No. of additional disorders, median (IQR)	2.0 (1.0–2.0)	1.5 (1.0–2.0)	0.51
Autoimmune enteropathy	16/37 (43)	7/42 (17)	0.01
Coeliac disease	2/37 (5)	12/42 (29)	0.008
Autoimmune thyroid disease	6/37 (16)	17/42 (40)	0.025
Autoimmune haematological disease^c^	5/37 (14)	6/42 (14)	1.00
Atopic dermatitis	6/37 (16)	5/42 (12)	0.75
Alopecia	0/37 (0)	3/42 (7)	0.24
Glomerulonephritis	6/37 (16)	0/42 (0)	0.008

Data are expressed as *n*/*n* (%) or median (range)

^a^Either the result of consanguineous union or from regions with a high rate of consanguinity as previously described [25]

^b^IPEX syndrome, caused by hemizygous mutations in *FOXP3*, is an X-linked recessive disorder and therefore only presents in males, hence the bias toward males in those with confirmed monogenic autoimmunity

^c^Thrombocytopenia, lymphoproliferative disease or hepatosplenomegaly

#### Type 1 diabetes controls

As previously described [12], we used control individuals from the Wellcome Trust Case Control Consortium (WTCCC) [14]. The 1963 individuals from the WTCCC had a clinical diagnosis of type 1 diabetes, were diagnosed before the age of 17 years and were treated with insulin from diagnosis.

### Ethics approval

The study was approved by the Genetic Beta Cell Research Bank, Exeter, UK with ethical approval from the North Wales Research Ethics Committee, UK.

### Genetic testing

We used targeted next-generation sequencing (NGS) as previously described [13] to test the seven genes known to cause monogenic diabetes with autoimmunity (*AIRE*, *IL2RA*, *FOXP3*, *LRBA*, *STAT1*, *STAT3*, *STAT5B*) in 79 individuals. All putative mutations were confirmed by Sanger sequencing or digital droplet PCR (primers available on request).

### Type 1 diabetes GRS

To generate a type 1 diabetes GRS we genotyped the top ten SNPs with the largest effect size as previously described, including both HLA and non-HLA regions [11, 12] (electronic supplementary material [ESM] Table 1) by targeted NGS, Sanger sequencing (primer sequences available on request) or the KASP assay (LGC, Teddington, UK).

### Antibody testing

Where available (*n* = 43), serum samples were prepared by the addition of 6.4 μl of 1 mol/l CaCl and 10 μl of 400 U thrombin to 250 μl EDTA plasma to induce clotting. Samples were then centrifuged for 7 min at 6000 *g* and the resulting supernatant fraction was removed for testing. GAD, insulin antigen-2 (IA-2) and zinc transporter-8 (ZnT8) antibody testing was performed using commercially available ELISA assays (RSR, Cardiff, UK) on the Dynex DS2 ELISA Robot (Dynex Technologies, Worthing, UK). Cut-offs for positivity are based on the 99th centile of 1500 controls [8]. The laboratory participates in the International Autoantibody Standardisation Programme.

### Statistical analysis

Logistic regression and receiver operating characteristic (ROC) curve analysis was used to assess the discriminatory power of biomarkers, clinical features and the type 1 diabetes GRS. Parametric (*t* test) and non-parametric (Mann–Whitney *U*) tests were used for continuous variables and Fisher’s exact test was used to compare categorical variables. Statistical analyses were performed in Stata 14 (StataCorp, College Station, TX, USA).

## Results

### Molecular genetics

A mutation in a known monogenic autoimmunity gene was identified in 47% (37/79) of the individuals with diabetes and ≥1 autoimmune disorder diagnosed before the age of 5 years; 25 male participants had a hemizygous mutation in *FOXP3*, eight individuals had recessively inherited mutations in *LRBA*, two had recessively inherited *IL2RA* mutations and two had heterozygous gain-of-function *STAT3* mutations. Twelve of these individuals have been reported previously [2, 3, 15]. The remaining 42 individuals had early-onset multiple autoimmunity but did not have a mutation in a known gene. The group of individuals with ‘unknown aetiology’ will either have a polygenic predisposition to diabetes and other autoimmune disease or a monogenic cause of autoimmunity (including diabetes) that has not been described to date.

### The type 1 diabetes GRS is lower in monogenic autoimmunity than in individuals with multiple autoimmune disease of unknown aetiology

**Fig. 1:**
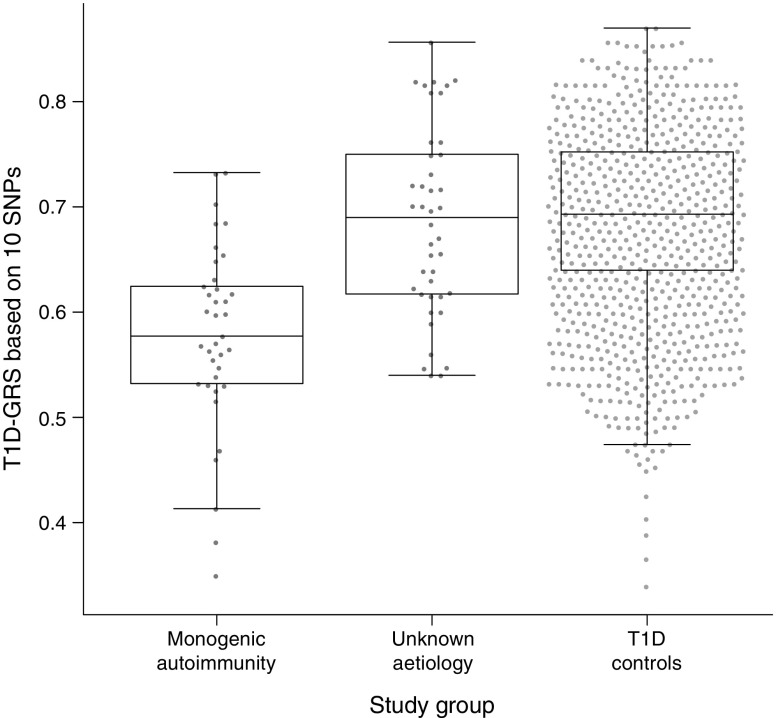
Boxplot of the type 1 diabetes GRS (T1D-GRS) in individuals with confirmed monogenic autoimmunity (*n* = 37), individuals with unknown aetiology (*n* = 42) and control individuals (*n* = 1963). The central line within the box represents the median and the upper and lower limits of the box represent the interquartile range. The whiskers are the most extreme values within 1.5× the interquartile range from the first and second quartiles. Those with confirmed monogenic autoimmunity had a lower median score than control individuals with type 1 diabetes (*p* < 0.0001), while those with unknown aetiology had a similar score to the controls (*p* = 0.63)

Individuals with confirmed monogenic autoimmunity had a markedly lower median type 1 diabetes GRS than those with early-onset autoimmunity of unknown aetiology (9th vs 49th centile of type 1 diabetes controls, *p* < 0.0001; Fig. 1). Individuals with unknown aetiology had a similar median type 1 diabetes GRS to that of the controls (49th vs 50th centile of type 1 diabetes controls, *p* = 0.63).

### The likelihood of identifying monogenic autoimmunity increases with decreasing type 1 diabetes GRS

**Fig. 2:**
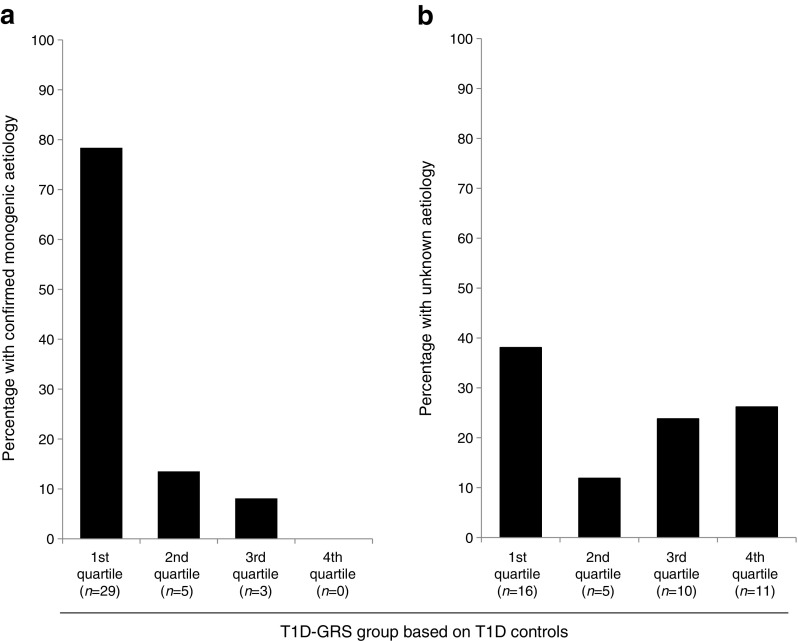
The type 1 diabetes GRS (T1D-GRS) in individuals with confirmed monogenic autoimmunity and individuals with unknown aetiology. (**a**) The proportion of individuals with confirmed monogenic autoimmunity (*n* = 37) in each quartile based on the scores for type 1 diabetes controls. The proportion of individuals with a confirmed monogenic cause was higher in individuals with a low T1D-GRS. (**b**) The proportion of individuals with early-onset multiple autoimmunity of unknown aetiology (*n* = 42) in each quartile based on the scores for type 1 diabetes controls. There is an over-representation of individuals with a low T1D-GRS, suggesting that there are novel monogenic causes remaining to be found in our cohort

When the entire cohort of 79 individuals was split into quartiles defined by the type 1 diabetes controls, the likelihood of identifying monogenic autoimmunity decreased as the type 1 diabetes GRS increased. Sixty-nine per cent (29/42) with a score below the 25th centile had a mutation in a known gene while 0% (0/11) with a type 1 diabetes GRS above the 75th centile had a mutation in a known gene (Fig. 2a). Seventy-nine per cent (11/14) of those below the fifth centile had a mutation in a known gene and 0% (0/8) above the 95th centile had a mutation in a known gene (data not shown).

### Most individuals with unknown aetiology are likely to have polygenic clustering of type 1 diabetes and additional autoimmunity

The 42 individuals who did not have a known cause of monogenic autoimmunity had a similar distribution between the four type 1 diabetes GRS quartiles as seen in type 1 diabetes controls (*p* = 0.38, Fig. 2b). This would fit with most of the individuals, in whom a known cause was not found, having polygenic type 1 diabetes. The 37 individuals with confirmed monogenic autoimmunity were most likely to have a low type 1 diabetes GRS: 78% (29/37) of those with monogenic autoimmunity were in the first quartile of type 1 diabetes GRS while none (0/37) were in the fourth quartile (Fig. 2a, *p* < 0.0001).

### Individuals with monogenic autoimmunity develop diabetes earlier than those with unknown aetiology and have broadly different clinical features

The clinical features of the individuals in the study cohort with and without a known cause of monogenic diabetes are shown in Table 1. Those with confirmed monogenic autoimmunity were typically diagnosed earlier than those with unknown aetiology (age 5 weeks [range 0–83] vs 36 weeks [range 1–258], *p* < 0.0001). A similar proportion of individuals had a positive result for at least one of anti-GAD, IA-2 or ZnT8 autoantibodies: 44% (8/18) with mutation and 44% (11/25) unknown aetiology, *p* = 1.00. When comparing individuals positive for ≥1 islet autoantibody (*n* = 19), the GRS was lower in those with monogenic autoimmunity: 0.558 (interquartile range [IQR] 0.528–0.613) vs 0.716 (IQR 0.670–0.819), *p* = 0.0005. Insulin dose and the median number of autoimmune features were similar.

Organ-specific disorders showed different frequencies in the two groups (Table 1, overall *p* = 0.0002). Individuals with monogenic autoimmunity were more likely to have autoimmune enteropathy (OR 3.8 [95% CI 1.3, 10.8], *p* = 0.01) or glomerulonephritis (OR 17.5 [95% CI 0.95, 323.0], *p* = 0.008) and less likely to have AITD and/or coeliac disease than individuals with autoimmunity of unknown aetiology (OR 5.3 [95% CI 1.8, 16.6], *p* = 0.001). The clustering of type 1 diabetes, coeliac disease and thyroid disease in those without a known cause of monogenic autoimmunity is likely to reflect the shared predisposition resulting from *HLA-DR3* for type 1 diabetes, thyroid disease and coeliac disease. Of the individuals with diabetes and AITD or coeliac disease, 22/29 (76%) of those with unknown aetiology and 3/8 of those with a monogenic aetiology carry at least one copy of *DR3* (ESM Table 2).

### A combination of clinical features and type 1 diabetes GRS is highly discriminative of monogenic autoimmunity

**Fig. 3:**
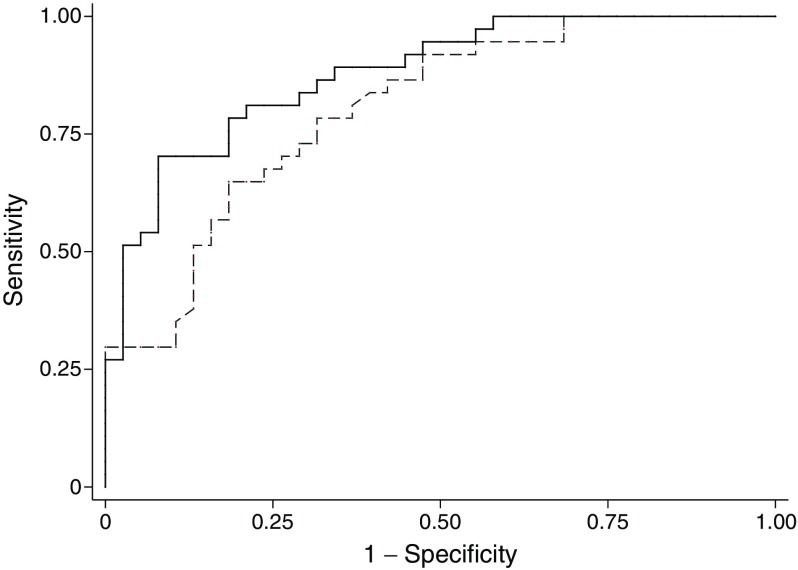
ROC curve, type 1 diabetes GRS and type 1 diabetes GRS combined with age at diabetes diagnosis in the discrimination of individuals with monogenic autoimmunity from those with unknown aetiology (*n* = 79). The dashed line shows type 1 diabetes GRS (AUC 0.80 [95% CI 0.70, 0.90]) and the solid line shows type 1 diabetes GRS combined with age at diabetes diagnosis (AUC 0.88 [95% CI 0.80, 0.95]). For age of diabetes diagnosis alone (AUC 0.79 [95% CI 0.69, 0.90]) and the presence of autoantibodies (AUC 0.49 [95% CI 0.34, 0.65]), data not shown

The type 1 diabetes GRS was highly discriminatory for identifying those with monogenic autoimmunity vs those with unknown aetiology (Fig. 3). ROC curve analysis gave an AUC for the type 1 diabetes GRS of 0.80 (95% CI 0.70, 0.90). Age of diagnosis had similar ROC AUC (0.79 [95% CI: 0.69, 0.90], *p* = 0.91) and when these two features were combined the discrimination improved against the type 1 diabetes GRS alone (ROC AUC 0.88 [95% CI: 0.80, 0.95], *p =* 0.04).

## Discussion

We have shown that a type 1 diabetes GRS can be used to identify individuals most likely to have a mutation in a monogenic autoimmune gene and could be used to prioritise individuals for gene discovery studies and, in combination with clinical features, genetic testing. Individuals with confirmed monogenic autoimmune disease have a markedly lower type 1 diabetes GRS than those with isolated type 1 diabetes or type 1 diabetes associated with other autoimmune disease, even when both conditions are diagnosed at a very young age.

The type 1 diabetes-associated antibodies have no discriminatory value, being present both in individuals with and without monogenic autoimmunity. While pancreatic autoantibodies have been previously shown to be specific (>57%) and highly sensitive (>99%) for discriminating between type 1 diabetes and non-autoimmune monogenic diabetes [8], we did not observe this in our cohort as monogenic autoimmunity often leads to autoantibody production. When islet autoantibodies were present, the type 1 diabetes GRS was lower in those with confirmed monogenic autoimmunity than in individuals with an unknown aetiology (0.558 vs 0.716). There is evidence that autoantibodies to harmonin and villin are diagnostic markers for individuals with IPEX syndrome [16], although we were unable to test this in our individuals with hemizygous *FOXP3* mutations. C-peptide testing is useful for identifying type 2 diabetes and MODY from type 1 diabetes [17]. However, as monogenic autoimmunity results in destruction of the pancreatic beta cells it is unlikely to be useful in our group and we were unable to assay serum C-peptide in our individuals. The type 1 diabetes GRS (ROC AUC 0.80) gave similar discrimination of monogenic autoimmunity from unknown aetiology than clinical features (ROC AUC for age at diagnosis 0.79) and a combination of these two features gave the best discrimination (ROC AUC 0.88).

**Fig. 4:**
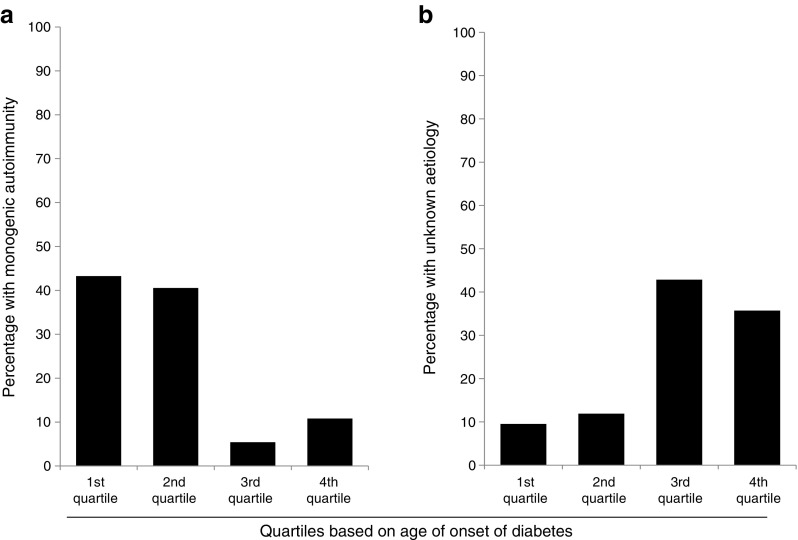
Individuals with confirmed monogenic autoimmunity and individuals with unknown aetiology were grouped in quartiles based on age at onset of diabetes: first quartile, 0–4 weeks; second 4–26 weeks; third 26–41 weeks; fourth 41–258 weeks. (**a**) Most individuals with monogenic autoimmunity were diagnosed in the first and second quartiles (43% and 41%, respectively), while a low proportion were diagnosed in the third and fourth quartiles (5% and 11%, respectively). (**b**) Most individuals with an unknown aetiology were diagnosed in the third and fourth quartiles (43% and 36%, respectively)

The overlap in clinical features may preclude their use for identifying individuals with monogenic autoimmunity. Age at diabetes onset was a good discriminator between the two groups of individuals; when split into quartiles based on age at diabetes diagnosis, 84% of those with monogenic autoimmunity were diagnosed in the first and second quartiles while 79% of those with an unknown aetiology were diagnosed in the third and fourth quartiles (*p* < 0.0001; Fig. 4). The range of age at diabetes diagnosis overlapped, however (monogenic autoimmunity 0–83 weeks; unknown aetiology 1–258 weeks), meaning it is less useful at an individual level. While autoimmune enteropathy and coeliac disease showed different prevalence in those with and without a mutation (Table 1), both groups included individuals with coeliac disease and autoimmune enteropathy. Furthermore, at the onset of symptoms these disorders can be difficult to distinguish clinically, particularly in very young individuals.

Those with confirmed monogenic autoimmunity were less likely to have AITD or coeliac disease in addition to type 1 diabetes than those with an unknown aetiology (22% vs 60%, OR 5.33). This is driven by the strong predisposing HLA allele *DR3* (through linkage with *DQ2*) in keeping with previous studies on shared HLA risk for these disorders [18]. The same effect does not appear to modulate disease in monogenic autoimmunity as none of the five individuals carrying the highest risk alleles for concurrent type 1 diabetes and coeliac disease—*DR3*/*DR3* and *DR3*/*DR4* [18]—had coeliac disease and only 3/14 with *DR3*/X had coeliac disease or AITD. Further study of a larger group of individuals is needed to confirm this effect as they may go on to develop coeliac disease or AITD later in childhood. We selected individuals with an extreme phenotype (diabetes and at least one autoimmune disease diagnosed before the age of 5 years), hence we found the extreme genotypes, both for monogenic and polygenic disease.

Interestingly, one individual in this study who had a type 1 diabetes GRS of 0.73 (65th centile of the type 1 diabetes controls) and was diagnosed with diabetes at the age of 3 weeks, was positive for GAD autoantibodies and had coeliac disease and AITD. Diabetes that presents extremely early suggests monogenic disease as >80% of individuals diagnosed before the age of 6 months have a mutation in a known gene [19]. However, their high genetic risk and positivity for GAD autoantibodies, along with the specific clinical manifestations, suggests that this may be a rare case of polygenic type 1 diabetes presenting in the neonatal period.

This study provides evidence that the polygenic risk of developing autoimmune diabetes does not affect the development of diabetes in individuals with monogenic autoimmunity. Previous reports of individuals with monogenic autoimmunity have shown that many do not develop diabetes (e.g. 70% of those reported to have gain-of-function *STAT3* mutations are not diabetic) [2, 20, 21]. The known risk alleles do not modify the phenotype in these individuals as the polygenic risk of developing autoimmune diabetes in our cohort of ‘with diabetes’ is similar to that in healthy controls (*p* = 0*.*162, data not shown). Further study of non-diabetic individuals with monogenic autoimmunity is warranted.

We propose that the type 1 diabetes GRS could be used to prioritise individuals for gene discovery studies. Our results suggest that a cut-off based on the 25th centile of type 1 diabetes controls would be suitable to guide selection of individuals for initial discovery as most individuals in this group have a monogenic cause. Furthermore, there was a small enrichment of individuals in the first quartile of the unknown-aetiology group (Fig. 2a), suggesting that some individuals in this group may have monogenic autoimmunity. These novel causes may be mutations in genes not previously associated with disease or deep-intronic/regulatory mutations in known genes. Identifying these novel aetiologies will further our understanding of the adaptive immune system and could provide new therapeutic targets as knowledge of the underlying pathway defect can allow personalised therapies. This is already happening for individuals with recessive *LRBA* mutations who can be treated with abatacept, which replaces the lost receptor molecule [22], and for individuals with IPEX syndrome who are amenable to haematopoietic stem cell transplantation, which if performed early can prevent the onset of organ-specific autoimmunity. Furthermore, identifying novel aetiologies will assist with research by preventing individuals with monogenic disease from taking part in clinical trials aimed at those with a polygenic aetiology.

The number of individuals with monogenic autoimmune disease available to study in our cohort was low (*n* = 37). However, to our knowledge, this is the largest series of individuals with monogenic autoimmune diabetes described in the literature to date. Interestingly, we did not identify any individuals with autoimmune polyendocrine syndrome type I due to biallelic *AIRE* mutations. The onset of autoimmune diabetes in this syndrome is typically later (age 30–50 years) [23, 24] and the specific clinically defining features, namely chronic mucocutaneous candidiasis and hypoparathyroidism, means identification may present less of a challenge.

Seven of the ten genotyped SNPs in this type 1 diabetes GRS cover loci that are associated (positively or negatively) with more than one autoimmune disease (ESM Table 1). However, some variants that predispose to multiple clinically distinct autoimmune disorders were not included in our panel. A recent meta-analysis of associations with childhood-onset autoimmune disease, including diabetes, identified 22 loci that associated with two or more of the disorders seen in our group of individuals [6]. A GRS tailored for regions with pleiotropic effects could offer higher discrimination of polygenic clustering of autoimmune disease and monogenic autoimmunity.

In conclusion, we have demonstrated that the type 1 diabetes GRS is useful for distinguishing clustering of early-onset type 1 diabetes with autoimmunity from monogenic autoimmune disease and could be used to prioritise individuals for gene discovery studies and follow-up genetic testing. Identifying these individuals might enable targeted treatment and would inform families and clinicians of the likely clinical course and increase understanding of the human immune system.

## Electronic supplementary material

ESM (PDF 93 kb)

## Data Availability

Study data is available on request from the corresponding author.

## Abbreviations

AITD: Autoimmune thyroid disease
GRS: Genetic risk score
IA-2: Insulin antigen-2
IPEX: Immunodysregulation, polyendocrinopathy, enteropathy, X-linked syndrome
IQR: Interquartile range
NGS: Next-generation sequencing
ROC: Receiver operating characteristic
WTCCC: Wellcome Trust Case Control Consortium
ZnT8: Zinc transporter-8

## References

[1] d’Hennezel E, Bin Dhuban K, Torgerson T, Piccirillo CA (2012). The immunogenetics of immune dysregulation, polyendocrinopathy, enteropathy, X linked (IPEX) syndrome. J Med Genet.

[2] Flanagan SE, Haapaniemi E, Russell MA (2014). Activating germline mutations in STAT3 cause early-onset multi-organ autoimmune disease. Nat Genet.

[3] Johnson MB, De Franco E, Lango-Allen H (2017). Recessively inherited *LRBA* mutations cause autoimmunity presenting as neonatal diabetes. Diabetes.

[4] Devendra D, Eisenbarth GS (2003). 17. Immunologic endocrine disorders. J Allergy Clin Immunol.

[5] Tosi R, Vismara D, Tanigaki N (1983). Evidence that celiac disease is primarily associated with a DC locus allelic specificity. Clin Immunol Immunopathol.

[6] Li YR, Li J, Zhao SD (2015). Meta-analysis of shared genetic architecture across ten pediatric autoimmune diseases. Nat Med.

[7] Batstra MR, Aanstoot HJ, Herbrink P (2001). Prediction and diagnosis of type 1 diabetes using β-cell autoantibodies. Clin Lab.

[8] McDonald TJ, Colclough K, Brown R (2011). Islet autoantibodies can discriminate maturity-onset diabetes of the young (MODY) from type 1 diabetes. Diabet Med.

[9] Tsuda M, Torgerson TR, Selmi C (2010). The spectrum of autoantibodies in IPEX syndrome is broad and includes anti-mitochondrial autoantibodies. J Autoimmun.

[10] Xavier-da-Silva MM, Moreira-Filho CA, Suzuki E, Patricio F, Coutinho A, Carneiro-Sampaio M (2015). Fetal-onset IPEX: report of two families and review of literature. Clin Immunol.

[11] Oram RA, Patel K, Hill A (2016). A type 1 diabetes genetic risk score can aid discrimination between type 1 and type 2 diabetes in young adults. Diabetes Care.

[12] Patel KA, Oram RA, Flanagan SE (2016). Type 1 diabetes genetic risk score: a novel tool to discriminate monogenic and type 1 diabetes. Diabetes.

[13] Ellard S, Lango Allen H, De Franco E (2013). Improved genetic testing for monogenic diabetes using targeted next-generation sequencing. Diabetologia.

[14] The Wellcome Trust Case Control Consortium (2007). Genome-wide association study of 14,000 cases of seven common diseases and 3,000 shared controls. Nature.

[15] Rubio-Cabezas O, Minton JA, Caswell R (2009). Clinical heterogeneity in patients with FOXP3 mutations presenting with permanent neonatal diabetes. Diabetes Care.

[16] Lampasona V, Passerini L, Barzaghi F (2013). Autoantibodies to harmonin and villin are diagnostic markers in children with IPEX syndrome. PLoS One.

[17] Ludvigsson J, Carlsson A, Forsander G (2012). C-peptide in the classification of diabetes in children and adolescents. Pediatr Diabetes.

[18] Smigoc Schweiger D, Mendez A, Kunilo Jamnik S (2016). High-risk genotypes HLA-DR3-DQ2/DR3-DQ2 and DR3-DQ2/DR4-DQ8 in co-occurrence of type 1 diabetes and celiac disease. Autoimmunity.

[19] De Franco E, Flanagan SE, Houghton JA (2015). The effect of early, comprehensive genomic testing on clinical care in neonatal diabetes: an international cohort study. Lancet.

[20] Haapaniemi EM, Kaustio M, Rajala HL (2015). Autoimmunity, hypogammaglobulinemia, lymphoproliferation, and mycobacterial disease in patients with activating mutations in STAT3. Blood.

[21] Milner JD, Vogel TP, Forbes L (2015). Early-onset lymphoproliferation and autoimmunity caused by germline STAT3 gain-of-function mutations. Blood.

[22] Lo B, Zhang K, Lu W (2015). Autoimmune disease. Patients with LRBA deficiency show CTLA4 loss and immune dysregulation responsive to abatacept therapy. Science.

[23] Husebye ES, Perheentupa J, Rautemaa R, Kampe O (2009). Clinical manifestations and management of patients with autoimmune polyendocrine syndrome type I. J Intern Med.

[24] Perheentupa J (2006). Autoimmune polyendocrinopathy-candidiasis-ectodermal dystrophy. J Clin Endocrinol Metab.

[25] Bittles AH (2008). A community genetics perspective on consanguineous marriage. Community Genet.

