# Supplementary material for: A type 1 diabetes genetic risk score can discriminate monogenic autoimmunity with diabetes from early-onset clustering of polygenic autoimmunity with diabetes
Source: Diabetologia. 2018 Feb 7;61(4):862–9. doi: 10.1007/s00125-018-4551-0 (PMC6448971; doi:10.1007/s00125-018-4551-0)
Supplement: Supplementary file 1 — (PDF 93 kb) [file 125_2018_4551_MOESM1_ESM.pdf]

A type 1 diabetes genetic risk score can discriminate monogenic autoimmunity with diabetes from early-onset clustering of polygenic autoimmunity with diabetes

Matthew B Johnson[1], Kashyap E Patel[1], Elisa De Franco[1], Jayne A L Houghton[2], Sian Ellard[1], Sarah E Flanagan[1], & Andrew T Hattersley[1]

[1]Insititure of Biomedical and Clinical Science, University of Exeter Medical School, Exeter, EX2 5AD, UK
[2]Molecular Genetics, Royal Devon and Exeter Hospital, UK

Supplementary table S1: SNPs used for the T1-GRS calculation. Disease associations taken from www.gwascatalog.com.

SNP(s)	Gene	Odds Ratio	Weight	Autoimmune disease associations
rs2187668, rs7454108	DR3/DR4-DQ8	48.18	3.87	Type 1 diabetes, Coeliac disease, Autoimmune thyroid disease, Autoimmune hepatitis
	DR3/DR3	21.12	3.05	
	DR4- DQ8/DR4-DQ8	21.98	3.09	
	DR4-DQ8/X	7.03	1.95	
	DR3/X	4.53	1.51	
rs1264813	HLA_A_24	1.54	0.43	Type 1 diabetes, Myasthenia gravis
rs2395029	HLA_B_5701	2.5	0.92	Type 1 diabetes, Psoriasis
rs3129889	HLA_DRB1_15	14.88	2.7	Type 1 diabetes (protective), Multiple sclerosis
rs2476601	*PTPN22*	1.96	0.67	Type 1 diabetes, Autoimmune thyroid disease Crohn's disease, Myasthenia gravis, Systemic lupus erythematosus, Rheumatoid arthritis
rs689	*INS*	1.75	0.56	Type 1 diabetes
rs12722495	*IL2RA*	1.58	0.46	Type 1 diabetes, coeliac disease, Systemic sclerosis
rs2292239	*ERBB3*	1.35	0.3	Type 1 diabetes
rs10509540	C10orf59	1.33	0.29	Type 1 diabetes

Supplementary table S2: HLA DR3 status of patients with coeliac disease and autoimmune thyroid disease in those with confirmed monogenic autoimmunity and unknown aetiology. X = any HLA allele other than DR3.

	**Monogenic autoimmunity (n=37)**			**Unknown aetiology (n=42)**		
	**Coeliac disease (n=2)**	**Autoimmune thyroid disease (n=6)**	**Either/both (n=8)**	**Coeliac disease (n=12)**	**Autoimmune thyroid disease (n=17)**	**Either/both (n=29)**
**DR3/DR3**	0	0	0	4	2	6
**DR3/X**	1	2	3	7	9	16
**X/X**	1	4	5	1	6	7
